# Synthesis of Lactulose in Continuous Stirred Tank Reactor With β-Galactosidase of *Apergillus oryzae* Immobilized in Monofunctional Glyoxyl Agarose Support

**DOI:** 10.3389/fbioe.2020.00699

**Published:** 2020-06-30

**Authors:** Claudia Ubilla, Nicolás Ramírez, Felipe Valdivia, Carlos Vera, Andrés Illanes, Cecilia Guerrero

**Affiliations:** ^1^School of Biochemical Engineering, Pontificia Universidad Católica de Valparaíso (PUCV), Valparaíso, Chile; ^2^Department of Biology, Faculty of Chemistry and Biology, Universidad de Santiago de Chile (USACH), Santiago, Chile

**Keywords:** β-galactosidase, lactulose, immobilization, prebiotic, agarose support, galacto-oligossacharides

## Abstract

Lactulose synthesis from fructose and lactose in continuous stirred tank (CSTR) reactor operation with glyoxyl-agarose immobilized *Aspergillus oryzae* β-galactosidase is reported for the first time. The effect of operational variables: inlet concentrations of sugar substrates, temperature, feed substrate molar ratio, enzyme loading and feed flow rate was studied on reactor performance. Even though the variation of each one affected to a certain extent lactulose yield (Y_*Lactulose*_), specific productivity (π_*Lactulose*_) and selectivity of the reaction (lactulose/transgalactosylated oligosaccharides molar ratio) (S_*Lu/TOS*_), the most significant effects were obtained by varying the inlet concentrations of sugar substrates and the feed substrate molar ratio. Maximum Y_*Lactulose*_ of 0.54 g⋅g^–1^ was obtained at 50°C, pH 4.5, 50% w/w inlet concentrations of sugar substrates, feed flowrate of 12 mL⋅min^–1^, fructose/lactose molar ratio of 8 and reactor enzyme load of 29.06 IU_*H*_⋅mL^–1^. At such conditions S_*Lu/TOS*_ was 3.7, lactose conversion (X_*Lactose*_) was 0.39 and total transgalactosylation yield was 0.762 g⋅g^–1^, meaning that 76% of the reacted lactose corresponded to transgalactosylation and 24% to hydrolysis, which is a definite advantage of this mode of operation. Even though X_*Lactose*_ in CSTR was lower than in other reported modes of operation for lactulose synthesis, transgalactosylation was more favored over hydrolysis which reduced the inhibitory effect of galactose on β-galactosidase.

## Introduction

Lactulose (4-O-ß-D-galactopyranosyl-D-fructose) is a synthetic disaccharide mainly used in the pharmaceutical field as a drug for the treatment of chronic constipation and hepatic encephalopathy (Schumann, 2002; Panesar and Kumari, 2011). Industrial production of lactulose is currently done by alkaline isomerization of lactose at elevated temperature. However, the chemical process has certain drawbacks, like the low yield attained and the formation of undesirable side products that make downstream operations for lactulose purification cumbersome and costly (Zokaee et al., 2002; Aider and de Halleux, 2007; Panesar and Kumari, 2011). Within this scenario, the production of lactulose by biocatalysis using enzymes from different microbial sources is a promising alternative for making the process more in line with green chemistry principles and sustainability (Foda and Lopez-Leiva, 2000; Mayer et al., 2010; Kim and Oh, 2012; Sitanggang et al., 2014, 2015; Guerrero et al., 2019). Most of the previous research has been focused on the search and evaluation of various biocatalysts, paying little attention to optimizing the reactor configuration. The most studied biocatalytic process for lactulose synthesis is the transgalactosylation of fructose with lactose catalyzed by β-galactosidase (β-G). However, this route is complex, since the enzyme is capable of transferring the galactosyl residue of lactose to any nucleophile bearing a hydroxyl group, even water. Therefore, transgalactosylation reactions compete with hydrolysis; besides, fructose, lactose and even lactulose can be acceptors of the galactosyl residue so that the product of synthesis is a rather complex mixture of monosaccharides (glucose and galactose), lactulose and transgalactosylated oligosaccharides (TOS) (Guerrero et al., 2015a; Rodrigues-Colinas et al., 2016; Vera et al., 2020). The relative rates of transgalactosylation and hydrolysis are strongly dependent on the enzyme origin and the substrates concentrations used in the reaction medium (Guerrero et al., 2015a; Vera et al., 2020). In this context, Guerrero et al. (2015a) reported that out of 11 commercial preparations of β-G evaluated, the one from *Aspergillus oryzae* produced the highest yield, productivity and selectivity of lactulose synthesis.

Several types of reactors and modes of operation have been studied (batch, repeated-batch, fed-batch, membrane, continuous packed-bed) with the purpose of increasing yield, productivity and selectivity of lactulose synthesis, using both soluble and immobilized β-G (Mayer et al., 2010; Song et al., 2012; Sitanggang et al., 2014; Guerrero et al., 2017). The highest yield, productivity and selectivity reported were obtained in batch stirred-tank reactors (BSTR) (Guerrero et al., 2017). Even though there is a number of reactor types and modes of operation to perform the reaction, the time distribution (RTC) and the kinetics of the reaction should be primary considerations to select the most adequate (Illanes, 2008a; Lindeque and Woodley, 2019). In this sense, continuous stirred tank reactor (CSTR) requires the same type of reactor vessel than BSTR, but now the operation is continuous, substrates being fed to the reactor and products being removed from it at the same rate, so that the reactor volume remains constant, and the enzyme is retained within the reactor during the whole operation. These features make CSTR an interesting alternative to BSTR for conducting lactulose synthesis.

Even though batch synthesis is easy to implement, it has the disadvantage of using a high amount of enzyme and the control of the reaction becomes problematic, since reaction kinetics should be precisely monitored (or modeled) in order to stop the reaction at the moment of maximum lactulose yield, before hydrolysis takes over (Kim and Oh, 2012; Hua et al., 2013; Sitanggang et al., 2014). Besides, considerable time is spent in the start-up and shutdown of a batch process, and time is also required for cleaning and preparation of the reactor for the next batch. So, the fraction of unproductive time in batch operation is significant (Lindeque and Woodley, 2019). On the contrary, synthesis of lactulose with immobilized β-G allows a better control of the reaction and facilitates product recovery and enzyme removal or reuse (Fernandez-Lafuente et al., 1998; Illanes, 2008b; Rodrigues et al., 2013). The fraction of unproductive time is significantly reduced with respect to BSTR operation due to the long-term continuous operation, which leads to a higher productivity. The properties of the biocatalyst are crucial in the operation of CSTR, since high operational stability and mechanical robustness are highly desirable to allow prolonged operation. Agitation is a key operational variable in CSTR that should properly balance good mixing to maintain the biocatalyst particles suspended and evenly distributed hopefully reducing the impact of diffusional restrictions, with biocatalyst integrity that will be threatened by the shear forces imposed by mixing (Illanes, 2008b).

Even though a large number of immobilized β-Gs has been reported, only few of them bring together the properties of high specific activity, mechanical robustness and operational stability (Lee et al., 2004; Mayer et al., 2010; Song et al., 2012; Guerrero et al., 2015b, 2017; Urrutia et al., 2018). Guerrero et al. (2017) made a comparative study of β-Gs immobilized in monofunctional an heterofunctional agarose supports, determining that multi-point covalent attachment to glyoxyl-agarose support produced a biocatalyst with high specific activity (2670 IU_*H*_⋅g^–1^) and operational stability, reporting a half-life of 2823 that corresponds to a stabilization factor (ratio of half-life of the immobilized enzyme and the soluble counterpart) of 29.4 at fructose-lactose molar ratio of 4 (Guerrero et al., 2017).

Even though several reactor configurations and modes of operation have been tested for the synthesis of transgalactosylated compounds, the use of CSTR for lactulose production with immobilized *A. oryzae* β-G has not been reported yet.

Based on the above background information, in this work the synthesis of lactulose and TOS derived from lactose and lactulose was studied in CSTR operation with immobilized *A. oryzae* β-G, taking advantage of the properties of this type of reactor and mode of operation (Illanes, 2008a; Woodley, 2015). The effect of temperature, feed sugars concentration, flowrate, substrates ratio and enzyme loading was evaluated on lactulose and TOS yields, productivities, selectivity and lactose conversion obtained in CSTR operation, comparing these results with those obtained with other modes of operation reported.

## Materials and Methods

### Materials

D (+) Lactose monohydrate, D (+) glucose, D (+) fructose, D (+) galactose, *o*-nitrophenol (*o-*NP), o-nitrophenyl-β-D-galactopyranoside (*o*-NPG) and galacto-oligosaccharides standards (3α-4β-3α galactotetraose, 4β-galactobiose) were supplied by Sigma (St Louis, MO, United States). Lactulose was provided by Discovery Fine Chemicals (Wimborne, United Kingdom). Agarose Bead Standard (6% cross-linked with epichlorohydrin) and packed bed reactor were purchased from Agarose Bead Technologies (Madrid, Spain). The enzyme used was Enzeco^TM^ Fungal Lactase Concentrate, a commercial preparation of *A. oryzae* β-galactosidase kindly donated by Enzyme Development Corporation, New York, NY, United States. All other reagents were analytical grade and supplied by Sigma or Merck (Darmstadt, Germany).

### Analysis

#### Determination of Enzymatic Activity of Biocatalysts

The enzymatic activity of the immobilized *A. oryzae* β-G catalysts was determined according to Guerrero et al. (2015b), defining one international unit of hydrolytic activity (IUH) as the amount of enzyme that hydrolyzes 1 μmol of o-NPG per minute at 45 mM o-NPG, pH 4.5 and 40°C.

#### Determination of Carbohydrates

A Jasco RI 2031 HPLC delivery system, provided with refractive index detector was used for the quantification of substrates (fructose and lactose) and products of the synthesis of lactulose (lactulose, TOS, galactose and glucose) according to the protocol reported by Guerrero et al. (2015b). The retention times were determined by checking the linear range of lactose, fructose, galactose, glucose, lactulose, 3α-4β-3α galactotetraose and 4β-galactobiose standards.

### Immobilization of *A. oryzae* β-Galactosidase in Glyoxyl Agarose Supports (GA)

Immobilization of *A. oryzae* β-G in monofunctional glyoxyl-agarose supports was done following the procedure described by Guisán (1988) and Guerrero et al. (2017). In order to determine the maximum hydrolytic potential of the biocatalyst, one international unit of hydrolytic activity (IU_*H*_) was defined as the amount of β-G that hydrolyzes 1 μmol of o-NPG per minute at 45 mM o-NPG, 40 °C and pH of 4.5 (Vera et al., 2011). The glyoxyl-agarose immobilized β-G had a specific activity of 3400 IU_*H*_⋅g ^–1^ as defined above.

### Synthesis of Lactulose in Continuous Stirred Tank Reactor (CSTR) With β-Galactosidase Immobilized in Glyoxyl-agarose Support

**FIGURE 1:**
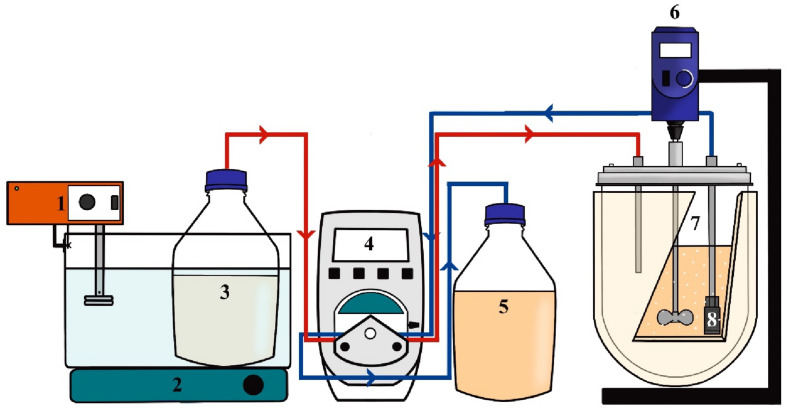
Experimental set-up for continuous stirred tank reactor operation in the synthesis of lactulose with *Aspergillus oryzae* β-galactosidase immobilized in glyoxyl-agarose support. 1: Heating immersion circulator, 2: Magnetic stirrer, 3: Substrate reservoir, 4: Pump, 5: Collector reservoir, 6: Rod stirrer, 7: Stirred tank, 8: Filter.

Figure 1 shows a schematic representation of the experimental system used for the synthesis of lactulose in CSTR. The reactor had an effective volume (V_*E*_) of 2 L, and the working volume for lactulose synthesis was 585 mL; temperature was kept at 50°C and pH was kept at 4.5. Sugar substrates were dissolved in 100 mM citrate-phosphate buffer pH 4.5 previously heated at 95°C and then cooled down to the reaction temperature. Different fructose/lactose molar ratios (F/L) were fed to the reactor using a Masterflex L/S 7525 (United States) pump and Masterflex 96400-14 silicone tubing connectors cured in peroxide, and 0.5 mL samples at the reactor outlet were taken every hour. Product distribution was determined by analyzing the amounts of lactulose, disaccharides, trisaccharides and tetrasaccharides produced. The assays were carried out in duplicate, with standard deviations always below 5%. Quantification of carbohydrates was carried out as described in See section “Determination of Carbohydrates.”

The effect of operation variables (inlet total carbohydrates, temperature, F/L, enzyme loading and flowrate) were evaluated in the synthesis of lactulose in CSTR operation in terms of the lactulose yield (Y_*L*__*actulose*_), TOS yield (Y_*TOS*_), total yield of transgalactosylation (Y_*T*_), lactulose total yield (Y_*Lu*_^∗^), specific productivity of lactulose synthesis (π_*L*__*actulose*_), lactose conversion (X_*L*__*actose*_) and selectivity of lactulose synthesis (S_*Lu/TOS*_) as previously described by Guerrero et al. (2017) for the batch synthesis of lactulose. These parameters are defined in Table 1.

**TABLE 1:** Parameters for the evaluation of lactulose synthesis in continuous stirred-tank reactor with β-galactosidase immobilized by multi-point attachment to glyoxyl agarose support.

**Continuous stirred tank reactor**		
**Parameters**	**Equation**	**Description**
Lactulose yield (Y_*Lactulose*_)	$Y_{L\,a\,c\,t\,u\,l\,o\,s\,e}=\frac{M_{L\,u}}{M_{L,i\,n}-M_{L,o\,u\,t}}$	Represents the fraction of the mass of reacted lactose (*M*_*L*,*in*_-*M*_*L*,*out*_) that is converted into lactulose at the maximum lactulose concentration attained in the reaction (M_*Lu*_).
Transgalactosylated oligosaccharides (TOS) yield (Y_*TOS*_)	${\text{Y}}_{\text{TOS}}=\frac{{\text{M}}_{\text{TOS}}}{M_{L,i\,n}-M_{L,o\,u\,t}}$	Represents the fraction of the initial mass of reacted lactose (*M*_*L*,*in*_-*M*_*L*,*out*_) that is converted into TOS (M_*TOS*_) at the maximum lactulose concentration attained in the reaction. TOS includes tri and tetra-saccharides formed from lactose or lactulose during the reaction.
Total yield of transgalactosylation reaction (Y_*T*_)	$Y_{T}=\frac{M_{T\,O\,S}+M_{L\,u}}{M_{L\,i\,n}-M_{L\,o\,u\,t}}$	Represents the fraction of the of the mass of reacted lactose (*M*_*L*,*in*_-*M*_*L*,*out*_) that is converted into lactulose and transgalactosylated oligosaccharides (tri and tetra-saccharides formed from lactose or lactulose during the reaction).
Lactulose total yield (Y_Lu_*)	*Y*_Lu_* = X_*L*_⋅Y_*Lu*_	Represents the fraction of the mass of total lactose that is converted into lactulose at the maximum lactulose concentration attained in the reaction (M_*Lu*_).
Specific productivity of lactulose synthesis (π_*Lactulose*_)	$\pi_{L\,a\,c\,t\,u\,l\,o\,s\,e}=\frac{M_{L\,u}}{M_{E}\cdot \tau \,}$	Represents the mass of lactulose produced per unit mas of protein in the enzyme preparation (M_*E*_) and unit of residence time (τ), evaluated at the maximum lactulose concentration obtained during the synthesis (M_*Lu*_).
Selectivity of lactulose synthesis (S_*Lu/TOS*_)	$S_{L\,u/T\,O\,S}=\frac{N_{L\,u}}{N_{T\,O\,S}}$	Represents the molar ratio of lactulose (Lu) to TOS in the reaction medium, evaluated at the maximum lactulose concentration obtained during the synthesis. N_*Lu*_ and N_*TOS*_ represent the moles of lactulose and TOS respectively.
Lactose conversion (X_*La*__*ctose*_)	$X_{L\,a\,c\,t\,o\,s\,e}=\frac{M_{L,i\,n}-M_{L,o\,u\,t}}{M_{L,i\,n}}$	Represents the mass fraction of lactose that enters the reactor, which is reacted during reactor operation.

#### Effect of Inlet Sugar Substrates Concentrations in the Synthesis of Lactulose in Continuous Stirred Tank Reactor (CSTR)

The effect of inlet sugar substrates concentrations (10, 20, 30, 40, 50, and 60% w/w) on Y_*Lactulose*_, Y_*TOS*_, Y_*T*_, X_*Lactose*_, π_*Lactulose*_, and S_*Lu/TOS*_ in the synthesis of lactulose in CSTR was evaluated at 50°C, pH 4.5, enzyme loading in the reactor of 29.06 IU_*H*_⋅mL^–1^ of reaction, flowrate of 6 mL⋅min^–1^ and F/L of 16.

#### Effect of Temperature in the Synthesis of Lactulose in Continuous Stirred Tank Reactor (CSTR)

The effect of temperature (50°C, 60°C, and 70°C) on Y_*Lactulose*_, Y_*TOS*_, Y_*T*_, X_*Lactose*_, π_*Lactulose*_, and S_*Lu/TOS*_ in the synthesis of lactulose in CSTR was evaluated at pH 4.5, 50% (w/w) inlet total carbohydrates, enzyme loading in the reactor of 29.06 IU_*H*_⋅mL^–1^, flowrate of 6 mL⋅min^–1^ and feed F/L of 16. CSTR was operated for 120 h at the different temperatures for determining the operational thermal stability of the biocatalyst. Inactivation profiles of the biocatalysts under operating conditions were modeled according to Henley and Sadana (1986).

#### Effect of the Molar Ratio of Fructose to Lactose in the Synthesis of Lactulose in Continuous Stirred Tank Reactor (CSTR)

The effect of F/L (4, 8, 16, and 24) on Y_*Lactulose*_, Y_*TOS*_, Y_*T*_, X_*Lactose*_, π_*Lactulose*_, and S_*Lu/TOS*_ in the synthesis of lactulose in CSTR was evaluated at 50°C, pH 4.5, 50% (w/w) inlet total carbohydrates, enzyme loading in the reactor of 29.06 IU_*H*_mL^–1^, flowrate of 6, 9, and 12 mL⋅min^–1^ and F/L of 4, 8, 16, and 24.

#### Effect of Enzyme Loading in the Synthesis of Lactulose in Continuous Stirred Tank Reactor (CSTR)

The effect of enzyme loading in the reactor (17.4, 29.06, and 58.12 IU_*H*_⋅mL^–1^) on Y_*Lactulose*_, Y_*TOS*_, Y_*T*_, X_*Lactose*_, π_*Lactulose*_, and S_*Lu/TOS*_ in the synthesis of lactulose in CSTR was evaluated at 50°C, pH 4.5, 50% (w/w) inlet total carbohydrates, flowrate of 6 mL⋅min^–1^ and F/L of 4, 8, and 16. Enzyme loadings of 17.4, 29.06, and 58.12 IU_*H*_⋅mL^–1^ correspond to catalyst mass to flowrate ratios (m_*cat*_⋅F^–1^) of 0.5, 0.8, and 1.6 g⋅min⋅mL^–1^, respectively.

#### Effect of Flowrates in the Synthesis of Lactulose in Continuous Stirred Tank Reactor (CSTR)

The effect of flowrate (3, 6, 9, and 12 mL⋅min^–1^) in the synthesis of lactulose in CSTR was evaluated at 50°C, pH 4.5, 50% (w/w) inlet total carbohydrates, F/L (4, 8, and 16) and enzyme loading in the reactor of 29.06 IU_*H*_⋅mL^–1^. Flowrates of 3, 6, 9, and 12 mL⋅min^–1^ correspond to catalyst mass to flowrate ratios (m_*cat*_⋅F^–1^) of 1.6, 0.8, 0.5, and 0.4 g⋅min⋅mL^–1^, respectively.

## Results and Discussion

### Effect of Inlet Concentrations of Sugar Substrates in the Continuous Synthesis of Lactulose in Stirred Tank Reactor

**FIGURE 2:**
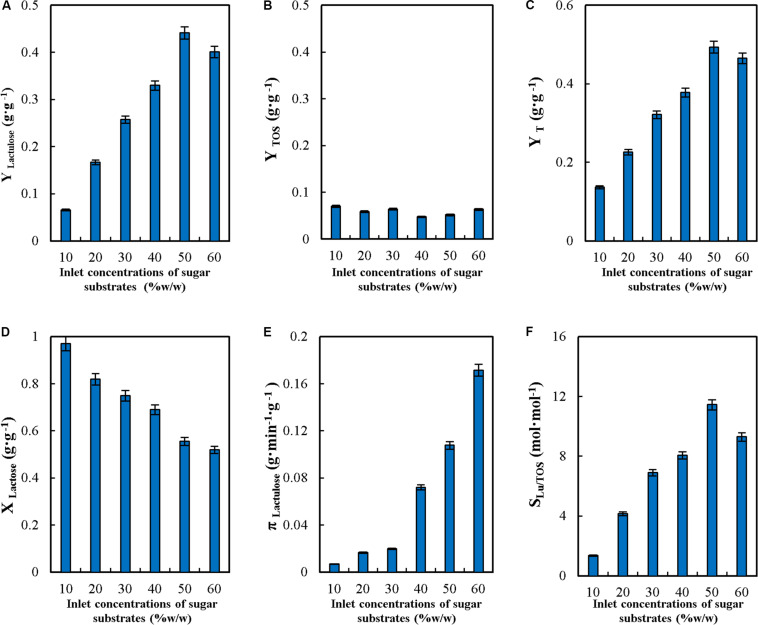
Effect of inlet concentrations of sugar substrates on yields (Y_*Lactulose*_, Y_*TOS*_ and Y_*T*_) **(A–C)**, lactose conversion (X_*Lactose*_) **(D)**, productivity of lactulose (π_*Lactulose*_) **(E)** and selectivity (S_*LU/TOS*_) **(F)** during the synthesis of lactulose with glyoxyl-agarose immobilized β-galactosidase from *A. oryzae* in continuous stirred tank reactor operation at pH 4.5, feed flowrate 6 mL⋅min^–1^, fructose/lactose molar ratio 16 and reactor enzyme load of 29.06 IU_*H*_⋅mL^–1^.

The concentration of the sugar substrates is one of the key variables in the synthesis of transgalactosylated compounds since, regardless of the enzyme used, concentrations over 30% favor transgalactosylation over hydrolysis (Vera et al., 2012). Guerrero et al. (2011), reported that lactulose concentration increased when increasing the initial total concentration of sugar substrates in batch, but yield and selectivity remained unchanged. Therefore, it is necessary to evaluate the effect of the inlet concentration of sugar substrates in the production of lactulose in CSTR. The effect on the operational parameters of synthesis was evaluated in the range from 10 to 60% w/w, as shown in Figure 2.

As seen in Figure 2, the increase in the inlet concentrations of sugar substrates between 10 and 50% (w/w) produced a continuous increase in Y_*Lactulose*_ (Figure 2A), and decreased at 60% (w/w), while Y_*TOS*_ remained constant in the whole range (Figure 2B). These results are different than those reported for the synthesis of lactulose with soluble *A. oryzae* β-G in batch, where no variation in these parameters was observed in the range from 40 to 60% (w/w) (Guerrero et al., 2011).

Y_*T*_ increased significantly with the increase in the inlet sugar substrates concentrations (Figure 2C), which denotes that transgalactosylation reactions are favored over hydrolysis as the concentration increases.

X_*L*_ decreased with inlet sugar substrates concentrations from a value close to 1 at 10% (w/w) down to 0.52 at 60% (w/w) (Figure 2D). These results highlight that at low inlet sugar substrates concentrations, hydrolysis is favored over transgalactosylation: even though 100% lactose reacted at 10% (w/w) only 13% of it went into lactulose and TOS synthesis, as reflected by Y_*T*_ (Figure 2C), while at 60% (w/w) only 52% of lactose reacted but 47% of it went into products of synthesis. These results are consistent with those reported for the synthesis of lactulose in batch (Guerrero et al., 2011) and for the synthesis of TOS (Vera et al., 2012).

Figure 2E shows that π_*Lu*_, increased with the increase in the inlet concentrations of sugar substrates, as a consequence of the higher concentration of lactulose produced. These results differ from reported for the synthesis of lactulose with the soluble enzyme, where π_*Lu*_ decreased by 18% when increasing the concentration from 50 to 60% (w/w) (Guerrero et al., 2011).

S_*Lu/TOS*_ increased with the increase in the inlet concentrations of sugar substrates (Figure 2F), which reflects that lactulose synthesis is favored over TOS (see the values obtained for Y_*Lactulose*_ and Y_*TOS*_).

In order to compare the value of Y_*L*__*actulose*_ with those reported for other enzymes and modes of operation, lactulose total yield (Y_*Lu*_^∗^) was defined (see Table 1) expressing the mass of lactulose produced per total mass of lactose fed into the reactor. The values obtained at different inlet concentrations of sugar substrates are shown in Table 2. Maximum Y_*Lu*_^∗^ was 0.246 g⋅g^–1^, obtained at 50% (w/w), being lower than the value of 0.31 g⋅g^–1^ reported for the soluble enzyme at a F/L of 12, and the value of 0.28 g⋅g^–1^ obtained with glyoxyl-agarose immobilized *A. oryzae* β-G under the same operational conditions in batch and in repeated batch operation (Guerrero et al., 2017); however, it is similar than the Y_*Lu*_^∗^ values of 0.254 g⋅g^–1^ and 0,254 g⋅g^–1^ obtained both with crosslinked aggregates (CLEAs) of the enzyme and with the enzyme covalently immobilized to the heterofunctional support amino-glyoxyl-agarose (Guerrero et al., 2015b, 2017). From the above results, the inlet concentrations of sugar substrates of 50% (w/w) was selected for the following experiments.

**TABLE 2:** Effect of operational variables (temperature, total initial sugars concentrations, reactor enzyme loading and feed flow rates) on lactulose yield with respect to total lactose reacted (Y_Lu_*) and lactulose concentration (C_*Lu*_) in the synthesis of lactulose in continuous stirred-tank reactor with glyoxyl agarose immobilized *A. oryzae* β-galactosidase.

**Molar Ratio of Fructose to Lactose**	**4**		**8**		**16**		**24**	
	Y_Lu_*	**C_*Lu*_**	Y_Lu_*	**C_*Lu*_**	Y_Lu_*	**C_*Lu*_**	Y_Lu_*	**C_*Lu*_**
					
	**(g_*L*__*u*_/g_*Lactose total*_)**	**(g/L)**	**(g_*L*__*u*_/g_*Lactose total*_)**	**(g/L)**	**(g_*L*__*u*_/g_*Lactose total*_)**	**(g/L)**	**(g_*L*__*u*_/g_*Lactose total*_)**	**(g/L)**
**Inlet concentrations of sugar substrates (% w/w)**								
10	−	−	−	−	0.064	4.15	−	−
20	−	−	−	−	0.137	7.51	−	−
30	−	−	−	−	0.193	8.39	−	−
40	−	−	−	−	0.228	10.52	−	−
50	−	−	−	−	0.246	9,19	−	−
60	−	−	−	−	0.209	7.27	−	−
**Temperature (°C)**								
50	−	−	−	−	0.246	9.14	−	−
60	−	−	−	−	0.185	7.79	−	−
70	−	−	−	−	0.162	0.53	−	−
**Reactor enzyme loading (IU_*H*_⋅mL^–1^)**								
17.4	0.143	28.99	0.182	22.20	0.208	13.93	−	−
29.06	0.149	30.19	0.193	23.60	0.246	16.43	−	−
58.12	0.171	34.58	0.221	26.89	0.259	17.37	−	−
**Feed flowrate (mL⋅min^–1^)**								
3	0.165	33.43	0.216	26.24	0.259	17.28	−	−
6	0.149	30.18	0.193	23.55	0.246	16.42	0.277	13.00
9	0.122	24.76	0.183	22.24	0.245	16.40	0.276	12.87
12	0.161	32.32	0.214	26.02	0.22	15.13	0.238	11.11

Lastly, Table 2 shows that the increase in the initial concentration of sugars produced an increase in the concentration of lactulose, which agrees with the results reported for the synthesis of lactulose with *K. lactis* β-galactosidase in a membrane reactor (Sitanggang et al., 2014). However, the magnitude of the concentrations of lactulose attained is not comparable, since the values of F/L were quite different, and this is critical since a higher proportion of fructose to lactose, even though favoring lactulose transgalactosylation over hydrolysis and TOS transgalactosylation, generates a lower concentration of lactose available for the formation of the enzyme-galactose transition complex, therefore reducing the concentration of lactulose attainable.

### Effect of Temperatures in the Continuous Synthesis of Lactulose in Stirred Tank Reactor

**FIGURE 3:**
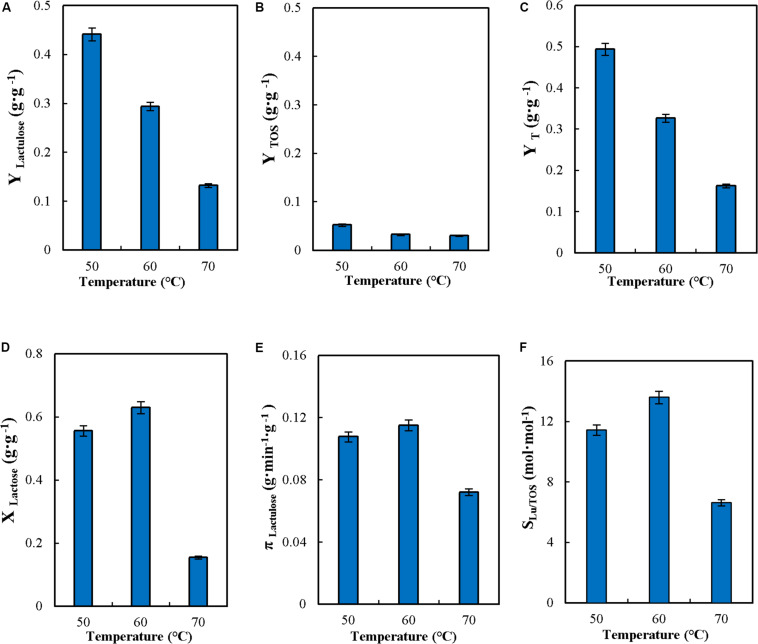
Effect of temperature on yields (Y_*Lactulose*_, Y_*TOS*_, and Y_*T*_) **(A–C)**, lactose conversion (X_*Lactose*_) **(D)**, productivity of lactulose (π_*Lactulose*_) **(E)** and selectivity (S_*LU/TOS*_) **(F)** during the synthesis of lactulose with glyoxyl-agarose immobilized β-galactosidase from *A. oryzae* in continuous stirred tank reactor operation at pH 4.5, 50% w/w inlet total concentrations of sugar substrates, feed flowrate 6 mL⋅min^–1^, fructose/lactose molar ratio 16 and reactor enzyme load of 29.06 IU_*H*_⋅mL^–1^.

The effect of temperature on CSTR performance was evaluated. High temperatures allow increasing the concentration of sugar substrates fed into the reactor (this is important for lactose, which is only moderately soluble), favor transgalactosylation over hydrolysis, and reaction rates are high; however high temperatures will promote subunit dissociation (Jurado et al., 2004; Vera et al., 2012) and in general will reduce enzyme stability (Illanes, 2008a; Urrutia et al., 2018). Therefore there is a compromise that needs to be solved to determine the best temperature to operate the reactor. Reaction was conducted in the range from 50 to 70°C as shown in Figure 3. The increase in temperature produced a significant decrease in Y_*Lactulose*_, Y_*TOS*_, Y_*T*_ and concentration of lactulose (Figure 3 and Table 2) similar to the data reported by Sitanggang et al. (2016) for the synthesis of lactulose with *A. oryzae* β-G in an enzymatic membrane reactor. However, these results differ from data reported for batch operation with the soluble *A. oryzae* β-G, where Y_*LU*_ and Y_*TOS*_ barely varied in the range from 40 to 60°C (Guerrero et al., 2011). The results obtained at 70°C may be explained by a more severe enzyme inactivation affecting the yield of synthesis (Figure 3).

X_*Lactose*,_ π_*Lu*_, and S_*Lu/TOS*_ increased slightly with the increase in temperature in the range between 50 and 60°C. Increase in π_*Lu*_ was also reported for the soluble enzyme, temperature increase producing an increase in the reaction rate of lactulose synthesis, therefore reducing reaction time (Guerrero et al., 2011).

**FIGURE 4:**
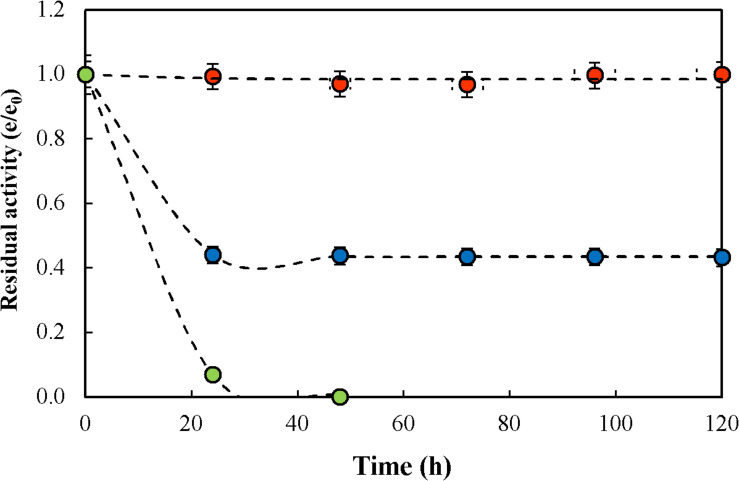
Thermal inactivation under reactive conditions of glyoxyl-agarose immobilized β-galactosidase from *A. oryzae* in citrate-phosphate buffer pH 4.5, fructose/lactose molar ratio of 16 and temperature of 50°C (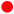), 60°C (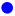) y 70°C (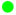). Dashed lines represent the inactivation kinetics according to the first-order one-stage inactivation mechanism with residual activity (see Eq. 1). Circles represent data points.

To the purpose of determining the thermal stability of the biocatalyst in continuous operation, a continuous run under reactive conditions in CSTR was conducted at F/L of 16. Results are presented in Figure 4 at 50, 60 y 70°C. As shown, no catalyst inactivation occurred at 50°C during the 120 h of continuous operation, but at 60 and 70°C inactivation was observed being higher at the latter temperature. Kinetics of thermal inactivation was modeled according to the deactivation mechanism proposed by Henley and Sadana (1986); one-stage first-order deactivation with residual activity was the one that better fitted the experimental results (equation 1).

(1) $$\frac{e_{0}}{e}=\alpha +\left(1-\alpha \right)\cdot e\,x\,p\,\left(-k_{D}\cdot t\right)$$

where (e_0_) and (e) are the initial and residual enzyme activity after time (t) respectively, the first-order inactivation rate constant (k_*D*_), the specific activity ratio of the final and initial enzyme species (α).

Based on the resulting model, k_*D*_, α and the catalyst half-life (t_1__/__2_) obtained are presented in Table 3. It can be appreciated that a 10°C change produced significant change in the operational stability of the biocatalyst. However t_1__/__2_ values are much higher than previously reported for the kinetics of enzyme inactivation under non-reactive conditions.

**TABLE 3:** Parameters of inactivation of glyoxyl agarose immobilized *A. oryzae* β-galactosidase at different temperatures, considering a one-stage first order mechanism with residual activity.

**Temperature (°C)**	**k_*D*_ (h^–1^)**	**α**	**t_1__/__2_ (h)**	**R^2^**
50	−	−	>120	−
60	0.189	0.436	21.8	0.99
70	0.112	0	13.4	1

*k_*D*_: first-order inactivation rate; α specific activity ratio of the final and initial enzyme species; R^2^: coefficient of determination.*

Urrutia et al. (2018) reported that inactivation kinetic at 60°C for β-Gs from *A. oryzae* was well described by a one-stage first-order mechanism of inactivation without residual activity, with t_1__/__2_ values of 0.43 h at 60°C. Bernal et al. (2013) compared the thermal stability of β-Gs from *A. oryzae* and *Bacillus circulans* under non-reactive conditions for the immobilization of glyoxyl agarose, reporting that the *A. oryzae* β-G was much more stable than the *B. circulans*β-G with a 3.6 times higher t_1__/__2_ at 60°C.

Huerta et al. (2011) reported the thermal inactivation of soluble *A. oryzae* β-G under non-reactive conditions obtaining a residual activity of 60% after 40 h at 50°C; however, the glyoxyl-agarose immobilized enzyme at the same conditions retained more than 80% of the initial activity.

When comparing enzyme inactivation at reactive conditions during lactulose synthesis, stability is strongly dependent on F/L. Guerrero et al. (2017) reported the inactivation of glyoxyl-agarose immobilized *A. oryzae* β-G during repeated batch operation at 50°C, obtaining a t_1__/__2_ of 2823 h, while under non-reactive conditions t_1__/__2_ was only 96 h, so that a stabilization factor of 29.4 was attained when operating at F/L of 4; however, at F/L of 20 t_1__/__2_ was 940 h, being the stabilization factor only 9.7, much lower than at a ratio of 4. Similar results were reported for CLEAs of *A. oryzae* β-G, where a t_1__/__2_ of 1070 h was obtained under reactive conditions and only 123 h at non-reactive conditions, obtaining a stabilization factor of 8.7 at F/L of 4; at F/L of 20 a t_1__/__2_ 315 h was obtained under reactive conditions, corresponding to a stabilization factor of 2.51, which is quite lower than obtained at lower ratios (Guerrero et al., 2015b, 2017). Even though, regardless of the substrate ratio used, the enzyme was always more stable under reactive than under non-reactive conditions, which is due to the protecting effect of the sugars at high concentrations, and was also observed in CSTR operation at F/L of 16 (Figure 4). However, at temperatures higher than 60°C, thermal inactivation of the enzyme prevailed over the protection effect of sugars. Therefore, 50°C was determined as the maximum allowable temperature to operate a CSTR for the synthesis of lactulose. Considering that the magnitude of stabilization was strongly dependent on F/L, it is to be expected that operating the CSTR at higher ratios a higher stability can be obtained.

### Effect of the Molar Ratio of Fructose to Lactose in the Synthesis of Lactulose in Continuous Stirred Tank Reactor

**FIGURE 5:**
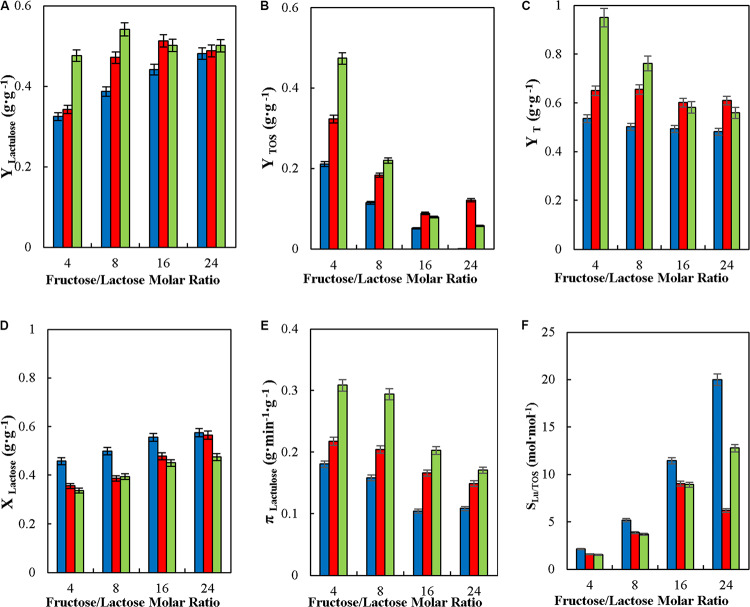
Effect of feed molar ratio of fructose to lactose on yields (Y_*Lactulose*_, Y_*TOS*_, and Y_*T*_) **(A–C)**, lactose conversion (X_*Lactose*_) **(D)**, productivity of lactulose (π_*Lactulose*_) **(E)** and selectivity (S_*LU/TOS*_) **(F)** during the synthesis of lactulose with glyoxyl-agarose immobilized β-galactosidase from *A. oryzae* in continuous stirred tank reactor operation, at 50°C, pH 4.5, 50% w/w inlet concentration of sugar substrates and reactor enzyme load of 29.06 IU_*H*_⋅mL^–1^, at feed flowrates of: 6 (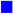), 9 (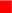), and 12 mL⋅min^–1^ (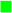).

Among the operational variables in lactulose synthesis, F/L is mostly important, since it was the one allowing significant variation in the selectivity of lactulose synthesis being progressively favored over the synthesis of TOS as F/L is increased. This has been reported already for the synthesis in batch with soluble and immobilized β-G and in CPBR with immobilized β-G (Guerrero et al., 2011, 2017, 2019). Therefore, the effect of this key variable was evaluated with respect to the operational variables in CSTR (Figure 5). This study was conducted at different feed flowrates, to determine if the substrates residence time in the reactor may influence the effect of feed flowrate in the values of the operational parameters.

Figure 5 shows that Y_*Lactulose*_, π_*Lactulose*_ and S_*Lu/TOS*_ increased significantly with the increase in F/L for all flowrate evaluated. Figure 5A shows that the increase in Y_*L*__*actulose*_ was higher at flowrates between 6 and 9 mL⋅min ^–1^ than at 12 mL⋅min ^–1^, where Y_*L*__*actulose*_ was relatively constant around 0.508 g⋅g^–1^. This effect of F/L on Y_*L*__*actulose*_, Y_*TOS*_ and Y_*T*_ agrees with results previously reported for the synthesis of lactulose in batch, in repeated-batch (Guerrero et al., 2017), in CPBR Guerrero et al., 2019) and in continuous membrane reactor operation (Sitanggang et al., 2016) with *A. oryzae*β-G, and also using *Kluyveromyces lactis* β-G in continuous membrane reactor operation (Sitanggang et al., 2015) and in batch (de Albuquerque et al., 2018).

Table 2 shows that the highest value of Y_*Lu*_^∗^, 0.277 g⋅g^–1^, was obtained at F/L of 24 and at a feed flowrate of 6 mL⋅min^–1^. This value is similar than the one reported in batch and repeated-batch with different biocatalysts; with β-G immobilized by covalent attachment to the heterofunctional support amino-glyoxyl-agarose and to the monofunctional support glyoxyl-agarose Y_*Lu*_^∗^ were 0.28 g⋅g^–1^ and 0.30 g⋅g^–1^ respectively at F/L of 20 (Guerrero et al., 2017). The higher concentration of lactulose of 14.17 g⋅L^–1^ was obtained at F/L of 4, which differs from Y_*Lu*_^∗^ whose maximum was obtained at F/L of 24 (Table 2). This is because at lower F/L the proportion of lactose is higher meaning that there are more galactosyl donor molecules available which is favors the formation of transgalactosylation products (lactulose and TOS); however hydrolysis is more severe at those F/L and then yields are lower. Similar results were reported for the synthesis of lactulose with both *K. lactis* y *A. oryzae* β-G in continuous membrane reactor where an increase in F/L produced a reduction of the lactulose concentration obtained (Sitanggang et al., 2015, 2016).

Figure 5D shows that X_*L*__*actose*_, in CSTR operation was barely affected by the substrates ratio and feed flowrates at the levels evaluated (Guerrero et al., 2019), while π_*Lactulose*_ decreased significantly with the increase in F/L at all flowrates analyzed, as shown in Figure 5E, quite different than results reported in CPBR, where π_*Lactulose*_ increased with F/L at all flowrates tested (Guerrero et al., 2019). However, π_*Lactulose*_ increased with feed flowrate which may be explained by transgalactosylation being favored over hydrolysis at lower residence times, with an increase in Y_*T*_ with the increase in flowrate at the three ratios evaluated (see Figure 5C).

As reported for other modes of operation, F/L is the variable determining the selectivity of the reaction (Figure 5F), lactulose being favored over TOS synthesis at high F/L.

### Effect of Enzyme Loading in the Continuous Synthesis of Lactulose in Stirred Tank Reactor

**FIGURE 6:**
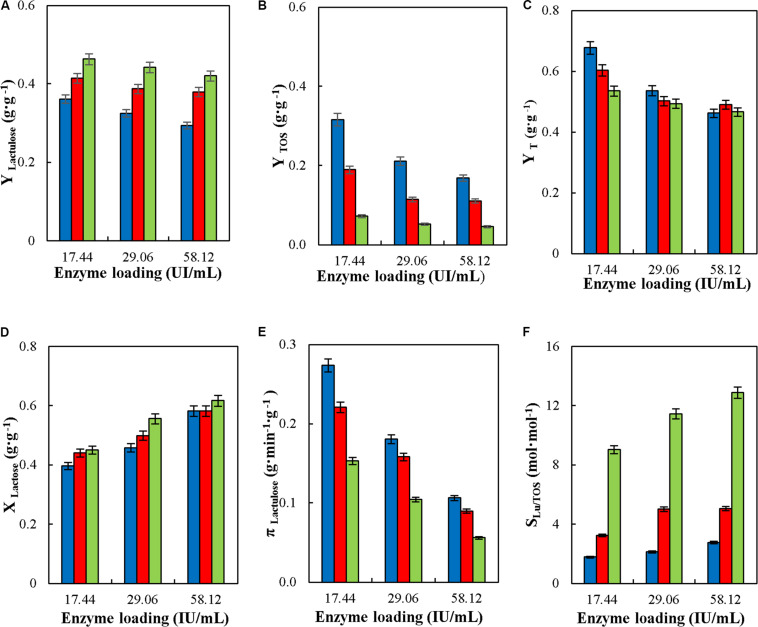
Effect of enzyme loading on yields (Y_*Lactulose*_, Y_*TOS*_, and Y_*T*_) **(A–C)**, lactose conversion (X_*Lactose*_) **(D)**, productivity of lactulose (π_*Lactulose*_) **(E)** and selectivity (S_*LU/TOS*_) **(F)** during the synthesis of lactulose with glyoxyl-agarose immobilized β-galactosidase from *A. oryzae* in continuous stirred tank reactor operation at 50°C, pH 4.5, 50% w/w inlet concentration of sugar substrates, feed flowrate 6 mL⋅min ^–1^ and fructose/lactose molar ratios of: 4 (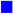), 8 (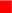), and 16 (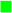).

Figure 6 shows the effect of reactor enzyme load on the operational parameters of lactulose synthesis in CSTR with glyoxyl-agarose immobilized *A. oryzae* β-galactosidase. Even though it was demonstrated that this variable did not affect yield or specific productivity in batch synthesis of TOS and lactulose with *A. oryzae* β-galactosidase and with enzymes form other sources (Lee et al., 2004; Guerrero et al., 2011; Vera et al., 2012), it did in CPBR synthesis of lactulose with glyoxyl-agarose immobilized *A. oryzae* β-galactosidase, where the decrease in reactor enzyme load produced an increase in Y_*Lactulose*_, Y_*TOS*_ and π_*Lactulose*_ (Guerrero et al., 2019). This agrees with the results obtained in CSTR, where the increase in reactor enzyme load reduced Y_*Lactulose*_, Y_*TOS*_, Y_*T*_ and π_*Lactulose*_ at all the F/L evaluated (Figures 6A–C,E). This may be because high enzyme loads favor hydrolysis over transgalactosylation, which is supported by the increase in X_*lactose*_ (Figure 6D) without an increase in Y_*T*_, which actually decreased significantly at high reactor enzyme loads at all the F/L tested (Figure 6C). A similar effect was recently reported for the continuous synthesis of lactulose in a membrane reactor with *K. lactis* and *A. oryzae* β-G (Sitanggang et al., 2015, 2016), where higher enzyme concentrations produced a more pronounced effect on hydrolysis than on transgalactosylation.

However, if lactulose yields are recalculated in terms of the total lactose fed into the reactor (Y_*Lu*_^∗^), a slight increase in yield is observed with the increase in reactor enzyme load at any F/L (Table 2). In this way, the highest Y_*Lu*_^∗^ was obtained at 34,000 IU_*H*_ at F/L of 16, being 0.26 g⋅g ^–1^. This value is similar than obtained with the same biocatalyst in other modes of reactor operation (Guerrero et al., 2017).

The increase in reactor enzyme load and F/L produced an increase in S_*Lu/TOS*_, reaching a maximum value of 12.87 at 34,000 IU_*H*_ and F/L 16, which is similar than previously reported for lactulose synthesis in batch (Guerrero et al., 2017), but different than reported for the synthesis in CPBR where the increase in reactor enzyme load had no effect on S_*Lu/TOS*_.

Lastly, lactulose concentration increased slightly with the increase in enzyme load at all F/L tested (Table 2); however it decreased with the increase in F/L, which is due to the lower proportion of lactose. These results agree with those reported for the continuous synthesis of lactulose in a membrane reactor with *A. oryzae* β-G (Sitanggang et al., 2016).

### Effect of Flow Rates in the Continuous Synthesis of Lactulose in Stirred Tank Reactor

**FIGURE 7:**
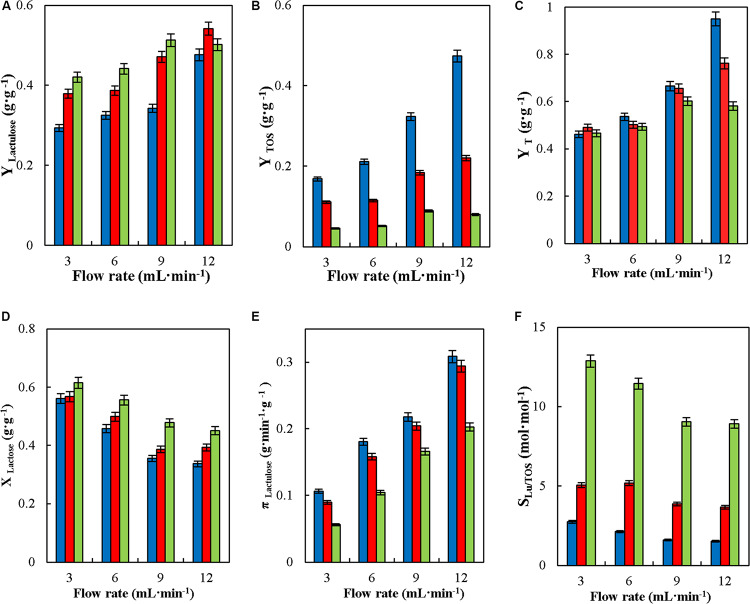
Effect of feed flowrate on yields (Y_*Lactulose*_, Y_*TOS*_, and Y_*T*_) **(A–C)**, lactose conversion (X_*Lactose*_) **(D)**, productivity of lactulose (π_*Lactulose*_) **(E)** and selectivity (S_*LU/TOS*_) **(F)** during the synthesis of lactulose with glyoxyl-agarose immobilized β-galactosidase from *A. oryzae* in continuous stirred tank reactor operation at 50°C, pH 4.5, 50% w/w inlet concentration of sugar substrates and reactor enzyme load of 29.06 IU_*H*_⋅mL^–1^, at fructose/lactose molar ratios of: 4 (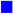), 8 (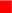), and 16 (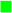).

Figure 7, shows the effect of feed flowrate on the operational parameters of lactulose synthesis in CSTR with glyoxyl-agarose immobilized β-G. Even though in continuous processes at constant reaction volume the variation in feed flowrate directly affects the value of hydraulic residence time (HRT), it has been demonstrated that it may also affect the concentration of product obtained (Sitanggang et al., 2015); therefore, the effect of this variable on CSTR operation needed to be determined. As seen the increase in feed flowrate produced an increase in Y_*Lactulose*_, Y_*TOS*_ and Y_*T*_ at the three F/L evaluated; however, the increase in Y_*TOS*_ was more pronounced at F/L of 4 (Figure 7B). This may be due to the higher faction of lactose in the reaction medium produced when increasing the feed flowrate at such F/L, which favors lactose to act as acceptor of transgalactosylated galactose, so increasing the synthesis of TOS. Similar results were reported for the synthesis of lactulose in CPBR with the same biocatalyst, where an increase in feed flowrate produced an increase in Y_*Lactulose*_ and Y_*TOS*_ (Guerrero et al., 2019). Song et al. (2013) reported that CPBR allowed obtaining higher Y_*Lactulose*_ than in BSTR when the former was operated at low flowrates; similar results were reported by Sitanggang et al. (2015), where high flowrates produced a slight decrease in Y_*Lactulose*_ in the continuous synthesis of lactulose in a membrane reactor with *K. lactis*β-G.

Values of Y_*T*_ remained constant at flowrate between 3 and 6 mL⋅min^–1^ at all the F/L tested, but an increase was produced at flowrates of 9 mL⋅min^–1^ and over for F/L of 4 and 8, while no significant variation in Y_*T*_ was observed at F/L 16 at all flowrates tested, meaning that at the latter there is no significant effect of feed flowrate on Y_*TOS*_. The highest Y_*T*_ were obtained at 12 mL⋅min^–1^ at all the F/L tested, which denotes that a major part of lactose was converted into transgalactosylated products, so that transgalactosylation prevailed over hydrolysis (Figure 7C).

Figure 7D shows that X_*Lactose*_ decreased with feed flowrate at all the F/L tested, which agrees with results previously reported for the synthesis of lactulose in CPBR with the same biocatalyst (Guerrero et al., 2019) and those obtained in a membrane reactor with *K. lactis* β-G (Sitanggang et al., 2015). Considering both X_*Lactose*_ and Y_*Lactulose*_, the yield of lactulose with respect to total lactose fed into the reactor (Y_*Lu*_^∗^) was determined, which decreased with the increase in feed flowrate, as shown in Table 2.

The reduction in Y_*Lu*_^∗^ with the increase in feed flowrate, was also reported by Song et al. (2012) in a microreactor with *A*. *oryzae* β-galactosidase. They explained that due to the short reaction time, mass transfer of the substrate into the immobilized enzyme molecules was reduced affecting both its transgalactosylation and hydrolysis. The reduction in X_*Lactose*_ (Figure 7D) has also been reported by Kang (2013) for the hydrolysis of lactose with *K. lactis* β-G immobilized in Duolite A568, and by Mayer et al. (2010) for the synthesis of lactulose in CPBR with *Pyrococcus furiosus* β-G immobilized in an anionic-exchange resin and in Eupergit C.

Figure 7E shows the effect of feed flowrate on π_*Laculose*_. The higher values of π_*Laculose*_ were obtained at the higher feed flowrates (lower HRT) at the three F/L tested. Maximum π_*Lac*__*t*__*ulose*_ of 0.31 g_*Lactulose*_⋅(min^–1^⋅g_*catalyst*_^–1^) was obtained at 12 mL⋅min^–1^ and F/L 4. These results agree with those reported by Sitanggang et al. (2015) for the operation of CSTR, where π_*Lactulose*_ increased at shorter HRT due to the higher amount of effluent produced.

S_*Lu/TOS*_ decreased with the increase in feed flowrate, this effect being more pronounced at F/L 16 (Figure 7F), since at this ratio lactulose synthesis prevailed over TOS, while the opposite occurred at lower F/L (Figure 7B). Similar results were reported for the synthesis of lactulose with *A. oryzae* β-G, in CPBR, where the increase in feed flowrate resulted in a significant reduction in S_*Lu/*__*TOS*_ (Guerrero et al., 2019), while Sitanggang et al. (2015) reported no significant variation of S_*Lu/TOS*_ with feed flowrate in continuous membrane reactor operation.

Lactulose concentration slightly decreased with the increase in feed flowrate and F/L, which agrees with the results reported for the continuous synthesis of lactulose in a membrane reactor with both *A. oryzae* and *K. lactis*β-G (Sitanggang et al., 2015, 2016).

### Comparison of the Continuous Performance of Lactulose Synthesis in Continuous Stirred Tank Reactor With Other Modes of Operation Reported

Table 4 summarizes the synthesis of lactulose with glyoxyl-agarose immobilized *A. oryzae* β-G under different modes of operation and reaction conditions (temperature, pH, feed or initial total sugars concentrations and F/L). It is worth mentioning that comparison is not completely fair, since even though the same operation conditions were used, flowrates and sizes of the continuously operated reactors were different. However, Table 4 allows putting into context the reported results on CSTR.

**TABLE 4:** Lactulose and TOS yields (Y_*Lactulose*_ and Y_*TOS*_), selectivity (S_*Lu/TOS*_) and lactose conversion (X_*Lactose*_) in the synthesis of lactulose with glyoxyl agarose immobilized *A. oryzae* β-galactosidase in BSTR CSTR and CPBR at 50°C, pH 4.5 and 50% (w/w) total sugars concentration.

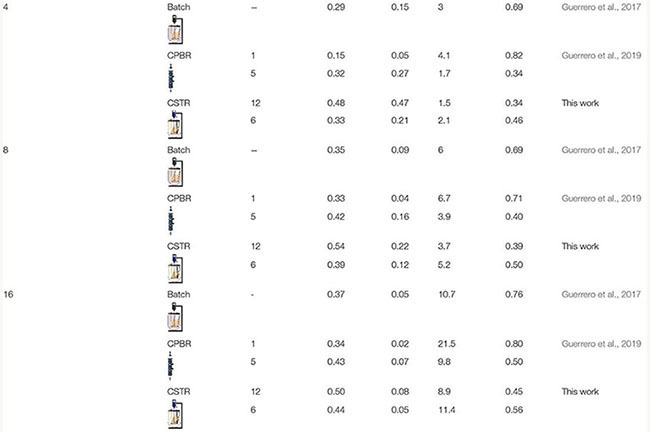

*Reactions in CPBR were conducted at a catalyst to packing inert material mass ratio of 1/8.*

Table 4, shows that at all the F/L evaluated, CSTR produced the highest Y_*Lactulose*_ and Y_*TOS*_; however, X_*Lactose*_ was the lowest. This means that CSTR allows reducing the hydrolysis of lactose, which is reflected in the higher Y_*Lactulose*_ and Y_*TOS*_ obtained, and also reduced the incidence of galactose competitive inhibition on β-G. In CPBR and BSTR, even though X_*Lactose*_ was high, Y_*Lactulose*_ and Y_*TOS*_ were low, which reflects the higher incidence of lactose hydrolysis, which is more severe at low feed flowrates (Guerrero et al., 2019). In this way, a clear advantage of CSTR is that transgalactosylation is privileged over lactose hydrolysis, which also alleviates the inhibitory effect of galactose (Vera et al., 2011; Song et al., 2013).

Table 4 shows that S_*Lu/TOS*_ are the lowest in CSTR, since it privileges not only lactulose synthesis but TOS as well, so that S_*Lu/TOS*_ are lower but Y_*T*_ are higher.

The results reported by Guerrero et al. (2017) in the synthesis of lactulose with glyoxyl-agarose immobilized *A. oryzae* β-G in repeated batch operation, show that increasing F/L increased the reduction of the specific activity of the biocatalyst after 10 batches, so that biocatalyst reuse is reduced by 80% at F/L of 16 with respect to 4. Similar results were reported for the synthesis of lactulose with CLEAs of β-G in repeated batch operation (Guerrero et al., 2015b). These results suggest a negative effect of high fructose concentrations. Even though the competitive inhibition of fructose on *K. lactis* β-G was reported (Song et al., 2013), it has also been suggested that fructose exerts a negative modulation on the stability of the biocatalyst, since at short reaction times no effect was observed on the activity of *A. oryzae*β-G (Guerrero et al., 2013). Therefore, whether inhibition or negative modulation of stability, the use of high fructose concentrations has a negative effect on lactulose synthesis, so that CSTR becomes an ideal mode of operation since the concentration of fructose in the reactor will be the lowest (if well mixed, the concentration inside the reactor will correspond to the outlet concentration), allowing to operate at rather high inlet F/L for favoring transgalactosylation over hydrolysis.

Results highlight that the production of lactulose in CSTR is a potentially viable strategy for developing an industrial process challenging the ongoing chemical synthesis of lactulose.

## Conclusion

Lactulose synthesis in CSTR with glyoxyl-agarose immobilized *A. oryzae* β-galactosidase proved to be a sound operational mode that compares favorably with conventional batch synthesis, since not only allows obtaining higher yield but also higher specific productivity thanks to the high operational stability of the immobilized enzyme. Among the variables studied, inlet concentrations of sugar substrates and feed fructose/lactose molar ratio were the ones having the most significant effect on the parameters of synthesis evaluated. Highest yield and productivity of lactulose synthesis in CSTR were obtained at 50°C, 29.06 IU_*H*_⋅ml ^–1^, 50% (w/w) inlet concentration of sugar substrates, feed flowrate 12 mL⋅min^–1^ and fructose/lactose molar ratios of 8, these values being higher than reported for the synthesis of lactulose in other modes of operation. Results reported in this work represent a first approximation for scaling up the enzymatic production of lactulose as an environmentally compliant alternative to its production by chemical synthesis.

## Data Availability Statement

The raw data supporting the conclusions of this article will be made available by the authors, without undue reservation.

## Author Contributions

CU, NR, and FV: conceptualization, investigation, and visualization. CV: conceptualization, methodology, writing – original draft preparation, and reviewing and editing. AI: conceptualization, methodology, writing – original draft preparation, and reviewing and editing. CG: conceptualization, methodology, formal analysis, resources, supervision, visualization, original draft preparation, and writing – reviewing and editing. All authors contributed to the article and approved the submitted version.

## Conflict of Interest

The authors declare that the research was conducted in the absence of any commercial or financial relationships that could be construed as a potential conflict of interest.
